# A Time Difference Method for Measurement of Phase Shift between Distributed Feedback Laser Diode (DFB-LD) Output Wavelength and Intensity

**DOI:** 10.3390/s150716153

**Published:** 2015-07-06

**Authors:** Yongning Liu, Jun Chang, Jie Lian, Zhaojun Liu, Qiang Wang, Cunguang Zhu

**Affiliations:** School of Information Science and Engineering, Shandong University, Jinan 250100, China; E-Mails: Liuyongning1990@163.com (Y.L.); lianjie@sdu.edu.cn (J.L.); zhaojunliu@sdu.edu.cn (Z.L.); iamwq1989@gmail.com (Q.W.); 2003410572@163.com (C.Z.)

**Keywords:** phase shift, DFB-LD, infrared spectroscopy, fiber optics sensors

## Abstract

A time difference method to conveniently measure the phase shift between output wavelength and intensity of distributed feedback laser diodes (DFB-LDs) was proposed. This approach takes advantage of asymmetric absorption positions at the same wavelength during wavelength increase and decrease tuning processes in the intensity-time curve by current modulation. For its practical implementation, a measurement example of phase shift was demonstrated by measuring a time difference between the first time and the second time attendances of the same gas absorption line in the intensity-time curve during one sine or triangle modulation circle. The phase shifts at modulation frequencies ranging from 50 Hz to 50 kHz were measured with a resolution of 0.001π. As the modulation frequency increased the shift value increased with a slowed growth rate.

## 1. Introduction

The features of a narrow frequency width and fast tunability make DFB-LDs extremely attractive in the areas of communications and optical sensors [1,2]. In most cases, the emission wavelength of a DFB-LD is tuned by an injection current at a faster tuning rate that can extend into the megahertz regime, when compared to the temperature tuning method. However, an unwanted optical-intensity modulation will accompany the change of injection current [3,4,5,6]. It was pointed out that output wavelength and intensity usually respond asynchronously to the fast-changing injection current, and wavelength lags behind intensity for most DFB-LDs [7]. Such a phase shift always brings a deviation in thedetected signal, such as the residual amplitude modulation noise in harmonic detection technology [7,8]. Therefore, to determine this shift is of great importance.

Li *et al.* [7] obtained a phase shift of 0.21π at frequency of 50 kHz with a fiber ring etalon at 1388 nm. Wang *et al.* [6] considered the phase shifts are almost the same at 20 kHz, 30 kHz and 40 kHz, about 0.09π, measured by a phase-generated carrier system with a telecommunication-grade laser at 1550 nm. Both the measurements were based on interferometry and obtained the relation between intensity and wavelength indirectly. However, the measured results can be easily affected by external interference.

Here we propose a convenient approach aimed at measuring such a phase shift mentioned above, which takes advantage of asymmetric absorption positions at the same wavelength but belonging to the wavelength increase and decrease tuning processes in the intensity-time curve with current modulation, respectively. A certain gas absorption line is selected to mark a wavelength in the intensity-time curve. Specifically, when absorption happens, it would reduce the intensity of the transmitted light; therefore, the information of tuning wavelengths can be reflected by such an intensity variation. What is more, within a full and symmetric current tuning circle, the same wavelength will appear twice, during the wavelength increase and decrease tuning processes, respectively, and if there exists a phase shift between output wavelength and intensity, the absorption positions will become asymmetric around the intensity symmetry axis in the transmitted intensity-time curve. The time points where absorption happens can be readily measured, and then the phase shift can be calculated based on the relation between the time points, which is explained in more detail in Figure 2. Herein we demonstrate the method by measuring the water vapor absorption line at 1368.6 nm with a full width at half maximum of about 0.0348 nm [9].The wavelength tuning range should be large enough in this measurement method to scan across the absorption line.

## 2. Basic Statement of the Measurement Method

**Figure 1:**
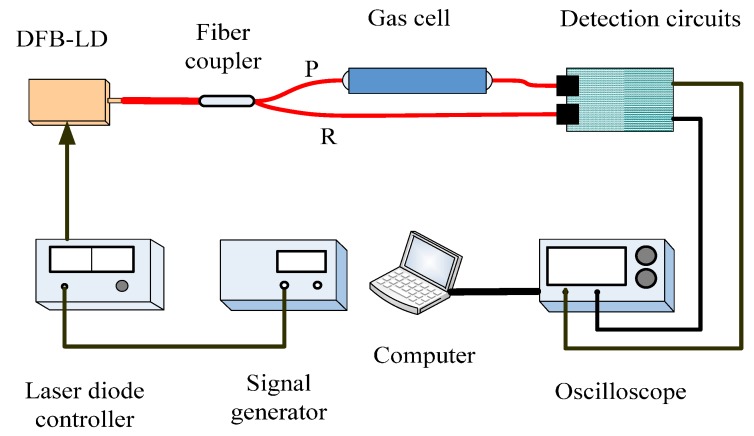
Structure diagram of TDLAS system: DFB-LD, distributed feedback laser diode; P, R, probe and reference light paths.

As shown in Figure 1, the measurement system is based on the dual-beam optical structure. A DFB-LD (model WSLS-137010C1424-20) operating at 1368.3 nm (at 28 °C with an injection current of 120 mA) is used as the light source, with emission characteristics of 0.1 mW/mA and 0.004nm/mA. A laser diode controller (LDC; model SRS LDC501) is employed to drive the LD with a resolution of 0.001 °C in temperature and a full scale accuracy of ±0.01% in current, which is connected to a signal generator for an external modulation signal with a conversion factor of 25 mA/V. The output light emitted from the DFB-LD is split by a wavelength flattened single-mode 1 × 2 fiber coupler into two paths (P path and R path) with a splitting ratio of 1:1. P path connects a 10-cmgas cell as a probe beam, while R path is used as a reference beam in assist. Light in both paths is detected by two identical InGaAs photo diodes (PDs) and processed with circuits in R minus P mode to get absorption peaks. After further amplification by circuits, the absorption peaks and R path signals are acquired by a digital oscilloscope for analysis with a computer program. The gas cell is placed in the air.

The DFB-LD is tuned with a sine wave in a large wavelength modulation range (covering the whole absorption line) at central wavelength of 1368.6nm. In view of the delay with respect to the injection current, the time dependent output intensity, wavelength and the phase shift between them can be expressed as:

(1) $$I(t)=\Delta I\;\text{sin}(2\pi ft+\varphi_{1})+I_{0}$$

(2) $$\lambda (t)=\Delta \lambda \;\text{sin}(2\pi ft+\varphi_{2})+\lambda_{c}$$

(3) $$\Delta \varphi =\varphi_{2}-\varphi_{1}$$

*I*(*t*) and λ(*t*) are the laser output intensity and wavelength; Δ*I* and Δλ are the modulation amplitudes; φ_1_ and φ_2_ are the phase delays of the injection current; λ_*c*_ is the central wavelength of the modulation range—ideally it should be the peak wavelength of the selected absorption line, and in implementation, we steadied this wavelength very closely around the ideal value by LDC, and *I*_0_ is average output intensity at λ_*c*_; Δφ is phase shift between output intensity and wavelength.

Target gas absorbs light following the Beer-Lambert law and the transmitted light intensity after gas cell will be:

(4) $$I_{t}(t)=I(t)\text{exp}\left\{-PS(T)NL\left[\frac{1}{2\pi }\frac{\Delta v_{L}}{{\left(\frac{1}{\lambda (t)}-\frac{1}{\lambda_{c}}\right)}^{2}+{\left(\frac{\Delta v_{L}}{2}\right)}^{2}}\right]\right\}$$

where *P* is the total gas pressure, *S*(*T*) is the line strength of the selected absorption peak, *N* is the mole fraction of the target gas, *L* is the gas absorption path length. The equation is based on a Lorentz line shape function with a full width at half maximum of Δυ_*L*_, about 0.0348 nm (0.186 cm^−1^) for water 1368.6nm line at normal temperature and pressure, totally included in 0.3 nm (1.6 cm^−1^) wavelength tuning range.

**Figure 2:**
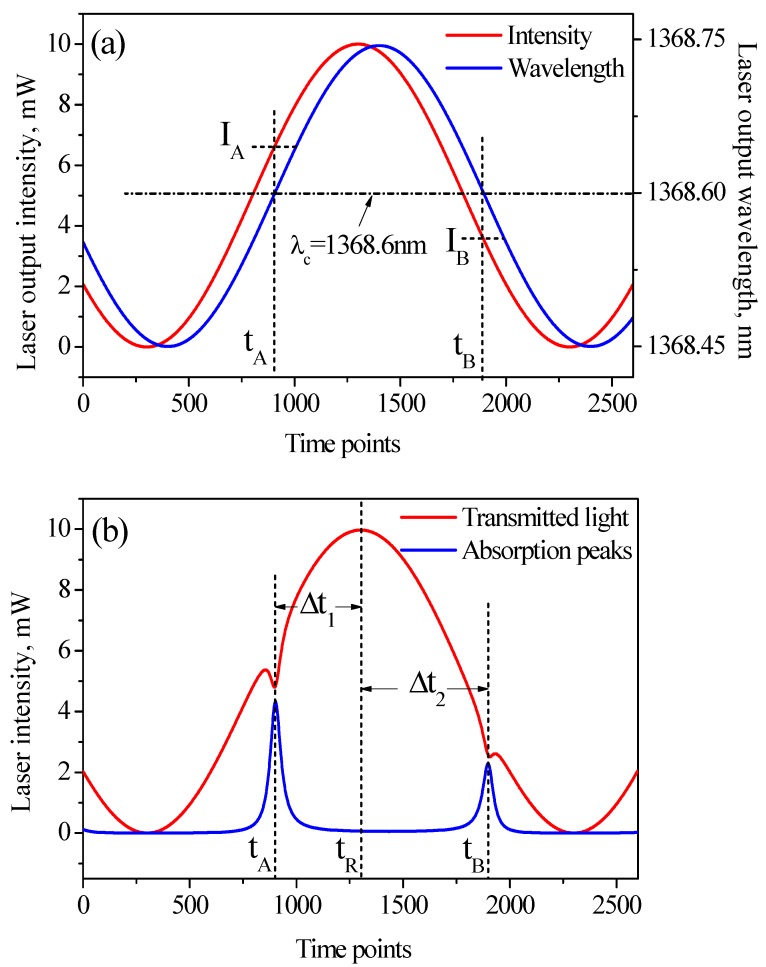
(**a**) DFB-LD output intensity (red) and wavelength (blue) with a phase shift of 0.1π; (**b**) Transmitted light intensity (red) and absorption peaks (blue).

Based on the above equations and conditions, we simulated how the phase shift affects the transmitted light in our system. Assuming that the output wavelength lags behind intensity by 0.1π (*i.e.*, Δφ *=* 0.1π). The time dependent output wavelength and transmitted light intensity would be that shown in Figure 2. The absorption around 1368.6 nm would appear at *t*_A_ and *t*_B_ on the rising edge and falling edge, respectively. The related instantaneous laser intensities at the two time points are *I*_A_ and *I*_B_, and obviously *I*_A_ *≠* *I*_B_ as is given in Figure 2a. Namely, the absorption at the same wavelength will happen on *I*_A_ and *I*_B_ as Figure 2b shows.

Therefore the phase shift finally gives rise to an asymmetry around the intensity symmetry axis (marked as *t*_R_) on the intensity-time curve with the absorption information. Inversely, we can measure a time shift based on this asymmetric signal:

(5) $$\begin{array}{ll} \Delta t & =\left(\Delta t_{2}-\Delta t_{1}\right)/2\\ & =\left(t_{A}+t_{B}-2t_{R}\right)/2 \end{array}$$

and the phase shift can be calculated as:

(6) Δφ=2πf·Δt

In practical measurements, the P path is subtracted from the R path signal and amplified by circuits to get clear absorption peaks, thus *t*_A_ and *t*_B_ can be estimated by extracting the maxima of absorption peaks accurately. *t*_R_ is estimated by using reference signal (R path), because absorption would influence the maxima extraction on transmitted intensity (P path) when absorption happens nearby.

## 3. Measurement Experiments and Results

**Figure 3:**
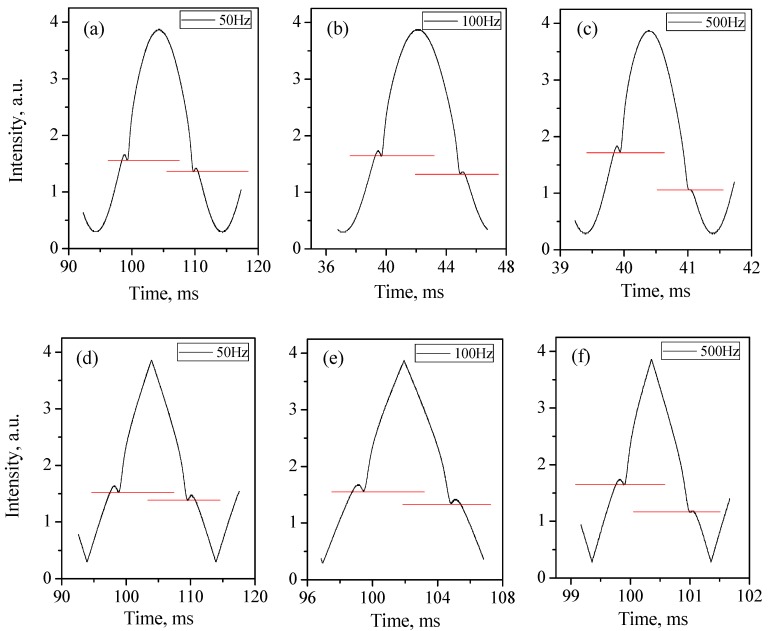
Transmitted intensity-time curves of sine and triangle modulation cases respectively at 50 Hz, 100 Hz and 500 Hz, specifically, (**a**) Sine wave modulation at 50 Hz; (**b**) Sine wave modulation at 100 Hz; (**c**) Sine wave modulation at 500 Hz; (**d**) Triangle wave modulation at 50 Hz; (**e**) Triangle wave modulation at 100 Hz; (**f**) Triangle wave modulation at 500 Hz.

In the following experiments, we adopted a sine wave and triangle wave as modulation waveforms, respectively. The generated wave amplitude is 3 V, providing a wavelength tuning range of about 0.3 nm for our DFB-LD. As the frequency is increased from 50 Hz to 500 Hz, the transmitted intensity asymmetry becomes greater, as Figure 3 shows.

**Figure 4:**
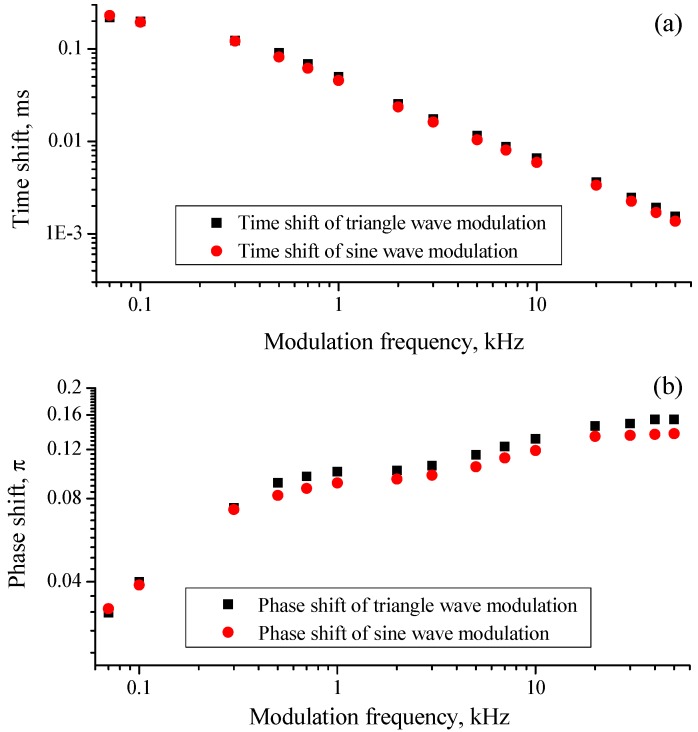
(**a**) Time shift of triangle wave modulation (black square) and sine wave modulation (red dot) at modulation frequencies from 50 Hz to 50 kHz; (**b**) Phase shift of triangle wave modulation (black square) and sine wave modulation (red dot) at modulation frequencies from 50 Hz to 50 kHz. All the axes are logarithmically scaled.

On the transmitted intensity-time curve, the absorption caused by the 1368.6-nm absorption line happened at different positions in the same modulation step, and the experiment data shown that as modulation frequency increased the phase shifts between output wavelength and intensity increased as well. Based on the above system and conditions, phase shifts between output intensity and wavelength were measured at modulation frequencies from 50 Hz to 50 kHz. For 1 kHz modulation, shift values were 0.1π and 0.091π, respectively, for the triangle wave and sine wave with a resolution of 0.001π. More measurement results are detailed in Figure 4. For both modulation waveforms, phase shifts increased with a slowed growth rate as modulation frequency increased. Shift value of triangle wave modulation was a little greater than that of sine wave at the same modulation frequency, shown in Figure 4b. This disparity should be on account of two reasons, one is the individual response of DFB-LD to the different current change rates of triangle and sine wave modulation forms, the other is the fact that triangle wave contains harmonics (fundamental frequency and its multiple frequencies) and the phase shift increases with frequency.

The accuracy of the phase shift measurement above depends on the parameters of the hardware system and data processing method used, while the performance of the analog circuits and data acquisition (digital oscilloscope) play a decisive role. Specifically, measurement accuracy of three time points (*t*_A_, *t*_B_, and *t*_R_) determines the system accuracy. However, the noise of each time point measurement is independent, referring to the relation in Equation (5), the total system noise may increase or decrease because of such independence and randomness. In view of the fact that measurement of *t*_B_ suffered the greater noise (absorption peak related to *t*_B_ will become weaker with frequency increases), we considered the measurement accuracy of *t*_B_ as the system accuracy superficially and discuss it particularly. For normal temperature and pressure, gas absorption line obeys Lorentz line shape, and the absorption can be expressed as:

(7) $$\begin{array}{ll} I_{\text{peak}}(t) & =I(t)-I_{t}(t)\\ & \approx G*I(t)*PS(T)NL*\frac{1}{2\pi }\frac{\Delta v_{L}}{{\left[v(t)-v_{c}\right]}^{2}+{\left(\frac{\Delta v_{L}}{2}\right)}^{2}} \end{array}$$

(8) $$v(t)=\frac{1}{\lambda (t)}$$

where *I*_peak_(*t*) is the intensity of absorption peaks, *G* is the circuit gain, and *v*(*t*) is the wavenumber. The measurement signal to noise ratio (*SNR*) of the above absorption peak is:

(9) $$SNR=\frac{I_{\text{peak}}}{I_{\text{noise}}}$$

(10) $$I_{\text{peak}}\approx G*I_{0}*PS(T)NL*\frac{1}{2\pi }\frac{\Delta v_{L}}{{\left(\frac{\Delta v_{L}}{2}\right)}^{2}}$$

where *I*_peak_ is the absorption peak maximum, and *I*_noise_ is the noise detected by the digital oscilloscope. Data points nearby the peak maximumat such a noise level allow theerror extraction by the program. Based on the above Equations (7)–(10) and the fact *I*(*t*) ≈ *I*_0_of the nearby peak, we can obtain an error wavelength range corresponding to the range of absorption peak intensity from *I*_peak_ − *I*_noise_to *I*_peak_, and express it as:

(11) $$\begin{array}{ll} \Delta \nu & =\nu -\nu_{c}\\ & \approx \sqrt{\frac{1}{SNR-1}}\frac{\Delta v_{L}}{2} \end{array}$$

To explain this more clearly, we define some parameters: in one complete modulation cycle (at frequency of *f*), the LD is tuned across a tuning range of *W* which can overlap the absorption line twice, and the absorption peaks in one complete modulation cycle are sampled to *N* points by the digital oscilloscope with a sampling rate of *S*. Taking Δυ as an intermediate parameter, the measuring accuracy can be finally defined as:

(12) $$\begin{array}{ll} \varphi_{0} & =2\pi \left[\Delta \nu /\left(\frac{W}{N}\right)\right]\frac{1}{S}f\\ & =2\pi \frac{N\Delta \nu }{SW}f\\ & =2\pi \frac{Nf}{SW}\sqrt{\frac{1}{SNR-1}}\frac{\Delta v_{L}}{2} \end{array}$$

and the theoretical resolution should be:

(13) $$\varphi_{R}=2\pi \frac{1}{S}f$$

Equation (12) indicates that the phase shift measuring accuracy is strongly decided by the signal to noise ratio of the intensity detection. In our experiment, the nominal oscilloscope sampling rate is 2 GS/s, while the actual sampling rate differed with the modulation frequency due to our options for choosing a suitable number of data points. Here,we take 50 kHz sinewave modulation as an example. For *N* = 2500, *f* = 50 kHz, *S* = 100 MS/s, *W* = 3.2 cm^−1^, *SNR* ≈ 41 (*I*_peak_ = 1.16 V, *I*_noise_ = 28 mV), Δυ_*L*_ = 0.186 cm^−1^, then the measurement accuracy in this case approximates 0.01π (the calculated result is φ_0_ = 0.011π). Meanwhile, with the same conditions, a triangle wave modulation at 50 kHz had a worse SNR of 32 (*I*_peak_ = 0.873 V, *I*_noise_ = 27 mV), and its measurement accuracy is calculated to be φ_0_ = 0.013π, bigger than that of the sine form. In practice, noise analysis should be more complex, and the measurement accuracy was a little worse than the calculated value. Additionally, good data processing step by theprogram, such as smoothing, should be very helpful for improving the accuracy.

## 4. Conclusions

In conclusion, a quite direct and easy method for measuring the phase shift between the output wavelength and intensity of DFB-LDs was proposed. The method takes advantage of asymmetric absorption positions at the same wavelength during the wavelength increase and decrease tuning processes in the intensity-time curve with current modulation, respectively. The shift can be calculated by measuring the time difference of the same absorption happening on the rising and falling edges of the transmitted intensity in the intensity-time curve. Based on this method, we measured such a phase shift by taking advantage of water absorption line at 1368.6 nm with our DFB-LD. For both sine wave and triangle wave modulation waveforms, the phase shift demonstrated a strong dependence on modulation frequency, and the shift value increased with a slowed growth rate as frequency increased from 50 Hz to 50 kHz, which is quite consistent with the statement in [7].According to the measured results in our work and [7], we can find that shift value varies with LD. It is likely that the active layers of LDs (operating at different wavelengths) respond differently to current change rates, which requires further study.
